# Microstructure and Magnetic Properties of Fe67.6-Pd32-In0.4 (at.%) Shape Memory Melt-Spun Ribbons

**DOI:** 10.3390/ma17071674

**Published:** 2024-04-05

**Authors:** David Vokoun, Yuan-Hung Lo, Oleg Heczko, Sneha Samal, Chen-Ti Hu

**Affiliations:** 1 FZU—Institute of Physics of the Czech Academy of Sciences, Na Slovance 1999/2, 182 00 Prague, Czech Republic; heczko@fzu.cz (O.H.); samal@fzu.cz (S.S.); 2 Department of Materials Science and Engineering, National Tsing Hua University, 101, Sec. 2, Kuang-Fu Road, Hsinchu 300044, Taiwan

**Keywords:** Fe-Pd shape memory alloy, martensitic transformation, multiferroic alloys, X-ray diffraction, magnetically induced strains

## Abstract

Fe-~30 at.%Pd is a ferromagnetic shape memory alloy (SMA) with a reversible thermoelastic fcc-fct phase transformation. The advantage of adding a small amount of Indium to Fe-Pd SMAs is, among other things, the upward shift of the transformation temperatures, which allows us to maintain the material in the martensitic state (fct structure) at room temperature. In this work, we study the microstructure and the magnetic properties of nominally Fe67.6-Pd32-In0.4 (at.%) melt-spun ribbons. Energy-dispersive spectroscopy analysis showed a certain level of non-uniformity of Indium distribution in the as-spun ribbon. However, the attempt to homogenize the ribbon by annealing at 1273 K for 120 h resulted in an unfavoured structural change to bct martensite. Magneto strains induced by a 9 kOe magnetic field reached over 400 ppm for certain field orientations, which is around four times more than the magneto strains of near-binary Fe-Pd shape memory alloys.

## 1. Introduction

The actuation of ferromagnetic shape memory alloys (FSMAs) under moderate magnetic field was first observed in the mid-1990s with Ni-Mn-Ga single crystals [1]. Since then, FSMAs have received much attention because of their ability to generate magnetically induced strain up to 10% [2,3] under external loads at maximum frequencies within the kilohertz range. The large magnetic strains and high actuation frequencies are achievable thanks to the rearrangement of martensite variants in an applied magnetic field [4]. Hence, it is an advantage to have martensite start temperature above room temperature.

Among all FSMAs, disordered Fe-~30 at.% Pd alloys have been studied extensively due to their good ductility and biocompatibility [5,6] of the material exhibiting (magnetic) shape memory effect [7,8], and also for their elastocaloric effect [9]. Magneto strains of Fe-~30 at.% Pd in a single crystal and a polycrystal can reach about 3% [10] and 60 ppm [11], respectively. An enhancement of magnetically induced strains (up to 800 ppm) for polycrystalline Fe-~30 at.% Pd prepared via a specially designed melt-spinning technique to provide strong texture and appropriate magnetization distribution was demonstrated by Furuya at al. [12]. Yasuda at al. reported magnetically induced strains up to 450 ppm through microstructure control of the Fe-~30.5 at.% Pd alloy [13].

It is well known that the fcc-fct transformation temperatures of Fe-~30 at.% Pd FSMAs can be increased by reducing the Pd content [14]. However, reducing the Pd content also may result in the formation of the non-thermoelastic bct martensite, which degrades the shape memory properties of the alloys including the mobility of the twin interfaces of martensitic variants beneficial for generating large magneto strains [15]. The potential problems associated with increasing the transformation temperatures by reducing the Pd content can be circumvented by alloying with ternary elements. Then, a better adjustment of the transformation temperatures can potentially be achieved without forming non-thermoelastic bct martensite. Therefore, the influence of various ternary elements has been studied both experimentally and theoretically using the first-principle calculations. Surprisingly, in contrast to Ni-Mn-Ga alloys, it is observed that the transformation temperatures increase with the decreasing valence electron concentration, e/a [16]. For purely binary Fe-~30 at.% Pd FSMAs, the e/a ratio reaches values of around 8.6.

The influence of Ni [14,16], Co [16,17,18], Pt [17,19,20], Mn [21,22], Rh [23,24], Cu [21,25], In [26], Si [27], and Ga [28] on the properties of Fe-~30 at.% Pd-X alloys has been reported. The formation of bct martensite was found to be suppressed when alloying with Pt, Mn, Rh, or Cu. As for the transformation temperature change due to adding ternary elements, when Pd [Fe] was replaced in part by Pt [Si], the transformation temperatures were reduced [17,20,27], whereas adding Ni and Co showed minor to no influence in shifting the transformation temperatures. Besides the influence of Pt on the transformation temperatures of Fe-Pd-Pt, an increase in Young’s modulus was observed [20]. According to calculations in [19], an addition of Pt has the potential of increasing magneto-crystalline anisotropy. An addition of Cu increased the transformation temperatures to 358 K [25]. On the other hand, when Fe was replaced in part by Co, the transformation temperatures were reduced. A 2% addition of Rh resulted in more ordered structures of L1_0_ [23,24]. A small addition of Mn increased the transformation temperatures to as high as 371 K [22]. Moreover, the addition of In (<3 at.%) increased the transformation temperatures to above 300 K [26].

To our knowledge, no other authors reported any work related to Fe-~30 at.%Pd FSMAs with the addition of Indium. Various properties of ternary Fe-Pd-In alloys with other compositions (with no shape memory effect) were reported by Matsumoto et al. [29]. They studied the effect of various levels of miscibility among Fe, Pd, and In elements using the fact that indium and iron are immiscible [30]. In [31,32], the authors studied Heusler Pd-In-Fe FSMAs, which exhibit interesting magnetic properties and superelasticity. Unlike Fe-~30 at.%Pd FSMAs, the austenite phase of Heusler Pd-In-Fe FSMAs has an ordered structure (L2_1_).

In addition to alloying with a third element, manufacturing techniques can also have a positive effect on suppressing the formation of bcc/bct martensite. Generally, methods using rapid solidification such as the melt-spinning method can result in partial or full suppression of the non-thermoelastic martensite, depending on the velocity of spinning wheel in the case of the melt-spinning method [33]. Here, we combine both approaches adding small amounts of Indium to the material prepared by rapid solidification.

Other manufacturing methods for the preparation of polycrystalline Fe-Pd-based SMAs are the magnetron sputtering method [34,35], electro-deposition [36], and methods of additive manufacturing [37]. 

Adding Indium to Fe-Pd FSMAs offers a change in the use of Fe-Pd-In together with Indium Tin oxide (ITO). The ITO thin film is a transparent conducting layer with many commercial applications in photo-electronic purposes [38,39], which also could be used as the light wave guiding switch material. It is believed that the bonding strength between ITO/Fe-Pd-In will be better than that of ITO on top of other FSMAs. Future study aims to develop the growth process of ITO thin film and certain types of optical wave switch devices on the top of the Fe-Pd-In ribbon. The refraction property of ITO film and light wave path material could be then changed by the magneto strain of the bottom Fe-Pd-In ribbon whenever the external magnetic field was applied. The final goal would be switching the light wave path remotely with an applying magnetic field at room temperature or above. The other potential applications of Fe-Pd SMAs involve actuators [40], elastocaloric devices [9], and medical devices [41]. 

The crystal structure of Indium at room temperature is hexagonal, which is closer to the fcc structure of Pd than to the bcc structure of Fe. Therefore, Indium might be suitable to replace, in part, the Pd atoms in the solid solution. For small amounts of Indium, however, it might not be important which element is replaced by Indium. 

The properties of Fe-Pd-In with various compositions with a focus on the influence of the addition of Indium on the transformation properties were reported in [26]. 

The purpose of the present work is to extent the previous work focusing on the ribbon with 0.4 at.% Indium prepared by rapid solidification in an effort to decrease the amount of the bct non-thermoelastic martensite and to obtain a specific textured microstructure in order to enhance the thermo-mechanical and magnetic properties of the shape memory materials.

## 2. Materials and Methods

SMA with chemical composition Fe_67.6_-Pd_32_-In_0.4_ (at.%) was prepared via a single-roller melt-spinning technique [42]. Before melt-spinning, Fe-~30Pd-0.5In ingot (8~10 g) was prepared by the vacuum arc melting technique from high-purity Fe, Pd, and In pellets (~99.99%), which were weighed up to milligram precision. During the melt-spinning procedure, the ingot was induction-melted in the quartz crucible just before being ejected by pressurized Ar gas onto a copper roller with a linear velocity of 8.1 m/s. The molten alloy was then rapidly solidified. The fabricated ribbons were 40~60 μm thick and 4 mm wide; some of them had lengths greater than 150 mm. The chemical compositions of the as-spun ribbons were determined with an inductively coupled plasma atomic emission spectrometer (ICP-AES). It was found that the Indium concentrations of the ribbons were consistently below the designated values. This is attributed to evaporation of Indium during processing. Some of the as-spun ribbons were annealed in argon atmosphere at 1273 K 120 h. The as-spun and annealed ribbons are denoted in the current study as ribbon S and ribbon A, respectively.

The microstructure was first observed with an optical microscope, model NEOPHOT, equipped with a digital camera. The etching solution consisted of 20% HCl and distilled water. The phases of the ribbons were identified via X-ray diffraction (XRD), first at room temperature (Rigaku Geigerflex D/max B equipped with CuKα radiation source) and later at various temperatures for a narrower range of the diffraction angle (X-ray diffractometer Bruker D8) equipped with a Cu Kα radiation source and thermal chamber DSC350 for cooling down up to 173 K). The XRD for various temperatures was conducted in a temperature range from 173 K to 313 K. The transformation temperatures of the as-spun ribbons—martensite start, M_s_, martensite finish, M_f_, austenite start, A_s_, and austenite finish, A_f_—were determined as described in [26].

For electron microscopy observations, the central area of the ribbon was thinned in a double-jet polisher (30% solution of HNO_3_ in methanol at 233 K, 10–20 V) to create a hole with ultrathin edges. This solution was also used for preparation of FeAl samples for transmission electron microscopy (TEM) by other authors [43]. The thin foil was studied at room temperature using the first TEM (JEM 1200EX microscope operating at 120 kV). Later, the TEM samples were also observed using a scanning electron microscope (SEM) FEI QUANTA 3D equipped with an energy-dispersive spectroscopy (EDS) detector.

The magnetic properties of ribbon S were measured using PPMS (Physical Property Measurement System, Quantum Design) for the in-plane direction at a low temperature (10 K), room temperature (300 K), and above room temperature (370 K). The angular dependence of magnetization was measured by VSM Microsense up to 20 kOe at room temperature.

Measurements of magnetically induced strains were performed on samples for various tilt angles. Strain gauges were glued to the ribbons along the rolling direction. Then, the ribbons were sandwiched between two glass plates with a 0.7 mm slit (to prevent bending). The source of magnetic field was a Vibrating Sample Magnetometer (VSM, model 4500 of EG&G Princeton Applied Research) with a maximum field capability of 11 kOe at room temperature.

## 3. Results and Discussion

The grain growth within the as-spun ribbons was affected by the melt spinning process, in which the rapidly chilled wheel side (of the ribbon) in contact with the wheel surface contained fine grains. 

**Figure 1:**
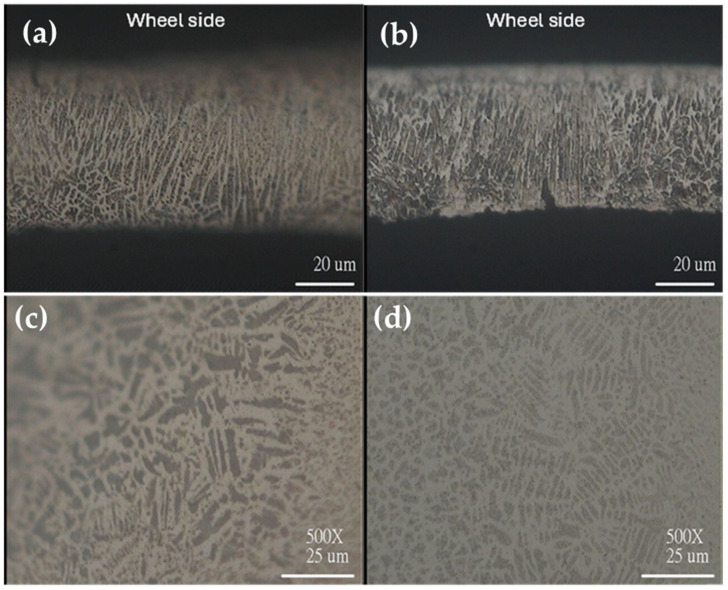

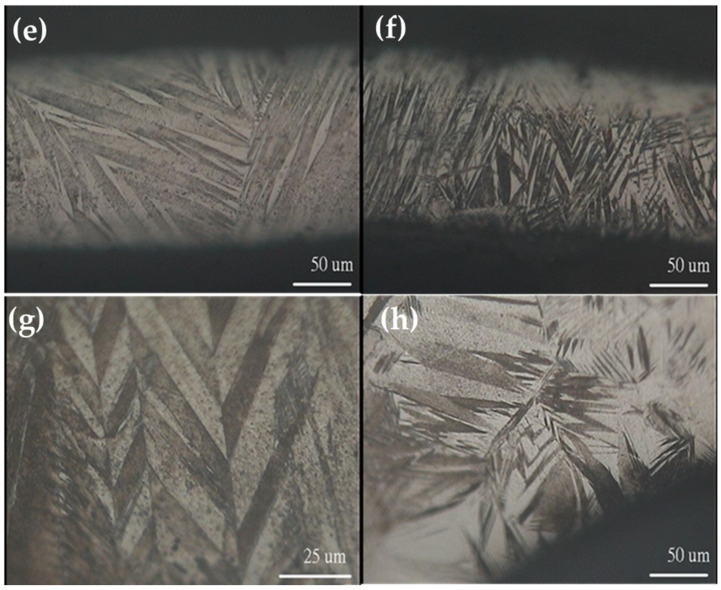
Optical micrographs of ribbon S: (**a**,**b**) cross-sections of two different positions, (**c**) free side, and (**d**) wheel side surface microstructures; annealed ribbon A: (**e**,**f**) cross-section, (**g**) free side, and (**h**) wheel side surface microstructures. For the cross-section micrographs (**a**,**b**,**e**,**f**) the wheel side is on the top as marked.

The free side exhibited coarser dendritic grains formed along the direction of heat flow, as shown in Figure 1a,b for the ribbon cross-section; the ribbon surface is shown in Figure 1c,d. Figure 1e–h shows optical micrographs of the annealed ribbons (ribbon A). The figure distinguishes between the cross-sectional and plain views. The microstructure of Ribbon A differs from that of ribbon S, and it is characterized by large visible martensite laminates of various orientations. This microstructure does not change on heating up to 373 K.

**Figure 2:**
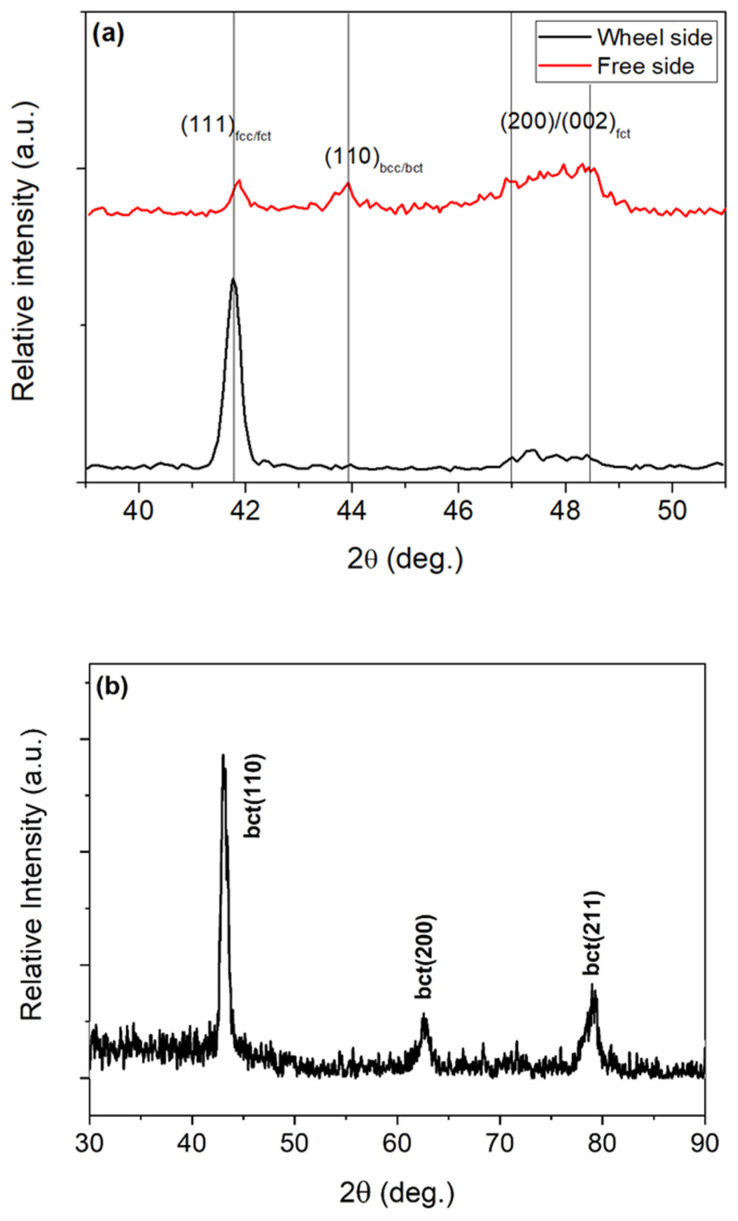
XRD (using Cu K_α_) patterns of as-spun ribbon S (**a**) and annealed ribbon A (**b**) at room temperature registered from the wheel side and the free side. For ribbon A, only the wheel side XRD pattern is shown.

Figure 2a shows the XRD patterns of the as-spun ribbon (S), registered from either the wheel or the free sides at room temperature. As the wheel side of the ribbon was chilled faster than the free side, it influenced the presence of the non-thermoelastic bct martensite (110). XRD shown in Figure 2a indicates a small amount of bct martensite on the ribbon’s free side, whereas on the wheel side the XRD pattern does not show any distinct peak belonging to the bct martensite. Importantly, both sides of the ribbon exhibited the (200)/(002) split associated with fct martensite, indicating that the Ms temperature is above room temperature. Figure 2b shows the XRD pattern of the annealed ribbon (A). The pattern is sharply different compared to the as-spun ribbon and there is no difference between the XRD patterns taken from either side of the ribbon. The pattern corresponds to the bct martensite. This means that the chosen annealing resulted in the structural change from fcc/fct to bct martensite present at room temperature. This martensite is not thermoelastic and is stable in a large temperature range confirmed by optical microscopy observation.

**Figure 3:**
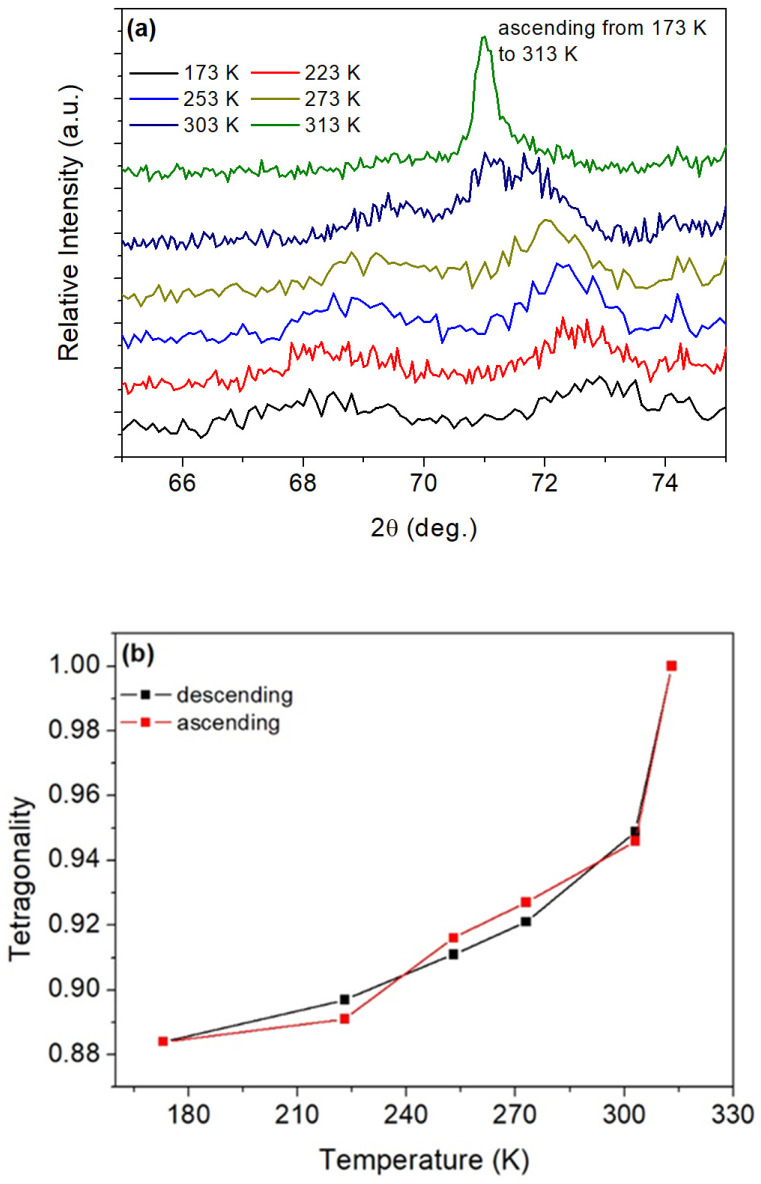
(**a**) The temperature dependent XRD (CuKα) patterns of as-spun ribbon S in the angle neighbourhood of (220) reflection during cooling from 373 K to 295 K, (**b**) derived tetragonality a/c as a function of temperature.

The XRD patterns of the free side of ribbon S were measured at various temperatures. On cooling, the fcc (111) diffraction peak (registered at 371 K) gradually shifted towards a higher angle and broadened as the material transformed to the fct martensite, whereas (200) and (220) fcc peaks each split into two separate peaks. Figure 3a shows, in detail, the splitting of the (220) peak with decreasing temperature. The broadening and peak splitting is an indication of the presence of the fct martensite. The tetragonality gradually changed during cooling/heating and reached a value of 0.88 at a temperature of 173 K (Figure 3b). The figure indicates a good reversibility of the fcc-fct martensitic transformation with a narrow hysteresis. The lattice parameters of Fe67.6-Pd32-In0.4 calculated based on the X-ray diffraction patterns were determined as follows: a_50_ = 0.3754 nm (austenite), a_-100_ = 0.3881 nm, c_-100_ = 0.3431 nm (fct martensite), and a_RT_ = 0.2912 nm (bcc martensite). The subscripts in the parameters denote temperature. The values reported above for Fe67.6-Pd32-In0.4 are close to the lattice parameter values of the corresponding phases of the neat Fe-Pd SMAs [7,13,44,45,46].

Based on the shape memory effect, the transformation temperatures of ribbon S were determined as follows: M_s_ = 311 K, M_f_ = 275 K, A_s_ = 277 K, and A_f_ = 319 K, confirming the XRD results. Despite narrow hysteresis the transformation of the ribbon is spread over broad temperature interval. It also follows from the temperature spread that at room temperature the ribbon was in mixed state of austenite and martensite. 

**Figure 4:**
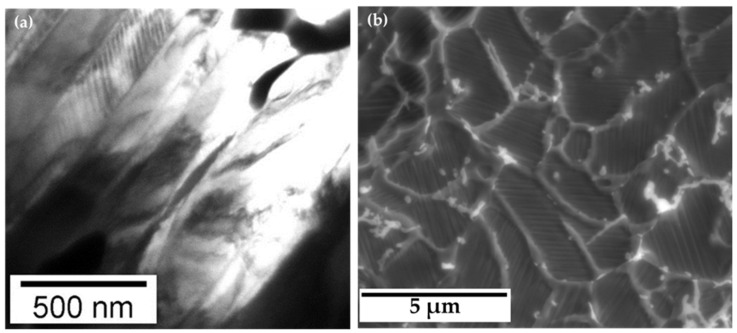
(**a**) Microstructure of as-spun ribbon (S) observed by TEM at room temperature, (**b**) a larger area (grain-like objects) observed by SEM.

Figure 4a shows a TEM image of the as-spun ribbon’s microstructure at a single grain level at room temperature. A finer (nanometric) martensite substructure is embedded between larger martensite plates of submicron size. A lower-magnification TEM image, not shown here, indicates that the size of the grainy objects is around a few micrometers. The SEM image taken from the TEM sample shows that most of the grainy objects have only a single orientation of the martensite plates and two arbitrary neighboring grains have often a different orientation of the martensitic plates (Figure 4b). Although the structure shown resembles grains, it cannot be ruled out that structural units other than grains are formed after rapid solidification. Furthermore, it is not clear what the white clusters in Figure 4b represent. To obtain more information about the white clusters, a compositional check through EDS was carried out in the area to be compared to the chemical composition of the other areas. The results of the composition analysis are summarized in Table 1. Interestingly, the white clusters are indium-rich and Fe-depleted. One explanation can be a possible preferential etching used for the fabrication of the TEM sample. This section may be divided by subheadings. It should provide a concise and precise description of the experimental results and their interpretation, as well as the experimental conclusions that can be drawn.

In conclusion, the white clusters formed as a part of the as-spun ribbon’s microstructure and visible in Figure 4b contain more indium than the area outside of the white clusters. The white clusters are mostly a part of the network which resembles thickened grain boundaries in Figure 4b. Importantly, these indium-rich white clusters might provide an enhanced bonding strength between ITO thin film and the Fe-Pd-In ribbon substrate in future study of remotely controlled light wave path devices as described in the Introduction.

**Figure 5:**
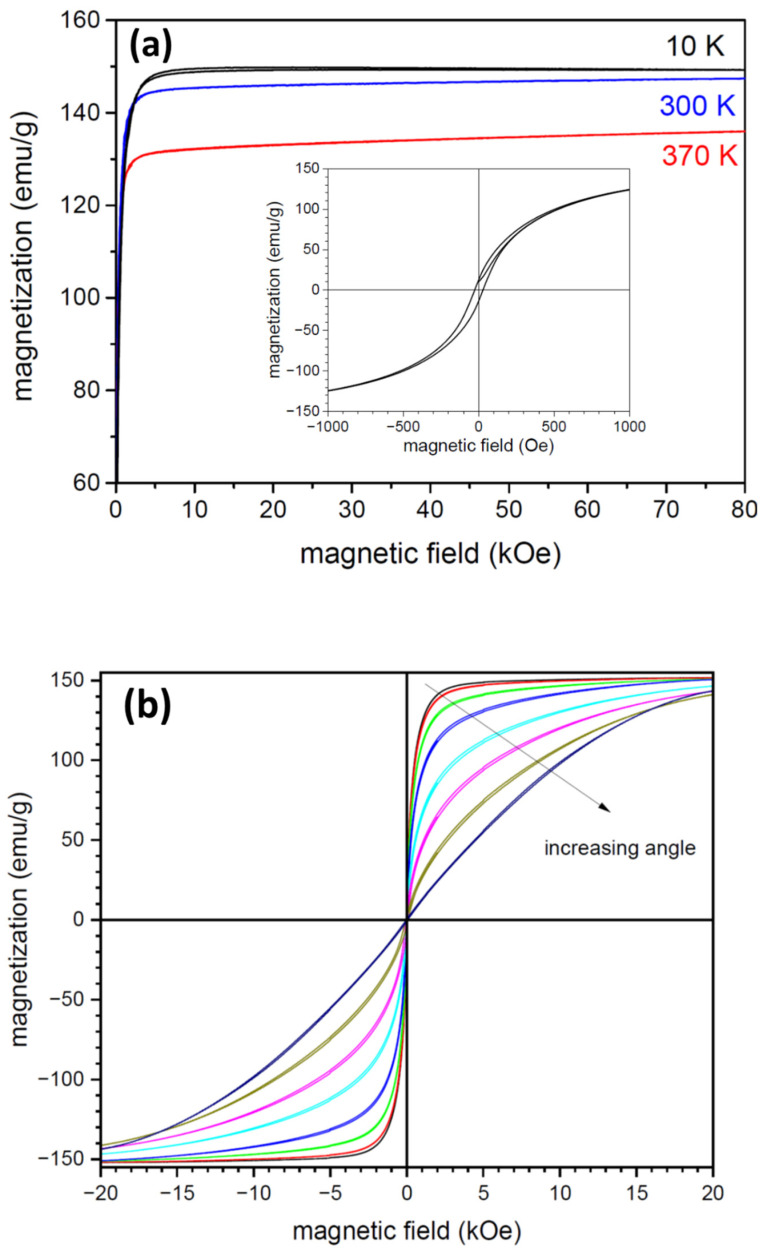
Magnetization curves of ribbon S: (**a**) at three temperatures (10, 300, 370 K) and a maximum magnetic field of 80 kOe, the in-plane direction was measured along the ribbon axis, inset with the detail of the magnetization (hysteresis) curve at low field measured at room temperature; (**b**) magnetization curves at room temperature for different angles between the ribbon plane and magnetic field. The angle increases as marked by arrow (curves for 0, 15, 30, 45, 60, 70, 80, 90 degrees).

In the following section, we will focus on magnetic and magnetoelastic properties of as-spun (S) and annealed (A) ribbon. Figure 5a shows the saturation of magnetization of ribbon S measured in plane and along the ribbon axis at three different temperatures. Only at 10 K is the sample magnetically saturated above 20 kOe (2 T). At room temperature and above, the magnetization curves shown in Figure 5a are not magnetically saturated for as high a magnetic field as 80 kOe. The relatively high slope can be due to heterogeneity of the sample and closeness of the Curie point. The saturation magnetization is 150 Am^2^/kg at 10K (1.93 μB) and decreases by about 2% at room temperature. The magnetization loop at 370 K denotes the austenite. From the curve difference between austenite and martensite, and assuming perfect columnar texture, we can estimate the magnetic anisotropy to be about 0.42 × 10^5^ J/m^3^, which is similar to the pure Fe-Pd alloy [20]. At room temperature, the difference is much smaller at about 0.13 × 10^5^ J/m^3^, which reflects the mixed state between austenite and martensite as determined by XRD. In contrast to the as-quenched ribbon of the other FSMA alloys such as Ni-Mn-Ga [47,48], the effect of the rapid solidification is not pronounced. The loops are saturated in a low field and exhibit low hysteresis even at 10 K, indicating that in-quenched stresses are not significant.

Figure 5b shows the magnetization curves of ribbon S measured at room temperature for the magnetic field in plane and when increasing the angle up to the field direction perpendicular to the surface. The maximum field was 20 kOe. The effect of the demagnetizing field for the in-plane field and for small angles can be neglected as the thickness of the other ribbon’s dimensions is small. In that case, the magnetization is saturated well below 20 kOe. The magnetization hysteresis is narrow, about 26 Oe for the in-plane magnetic field (see inset in Figure 5a), which is much less than the 125 Oe measured for Fe-29.9 at% Pd SMA for the as-spun ribbons [11]. For higher angles, the demagnetization increases, and the saturation field shifts to a higher field. In the perpendicular direction, the saturation was not reached even for 20 kOe. For increasing angles, the coercivity increases up to 87 Oe in a perpendicular direction. The magnetization loops measured in the ribbon plane (not shown) for various angles from along the perpendicular direction to the ribbon axis were nearly identical, revealing the magnetic homogeneity of the sample in plane. 

**Figure 6:**
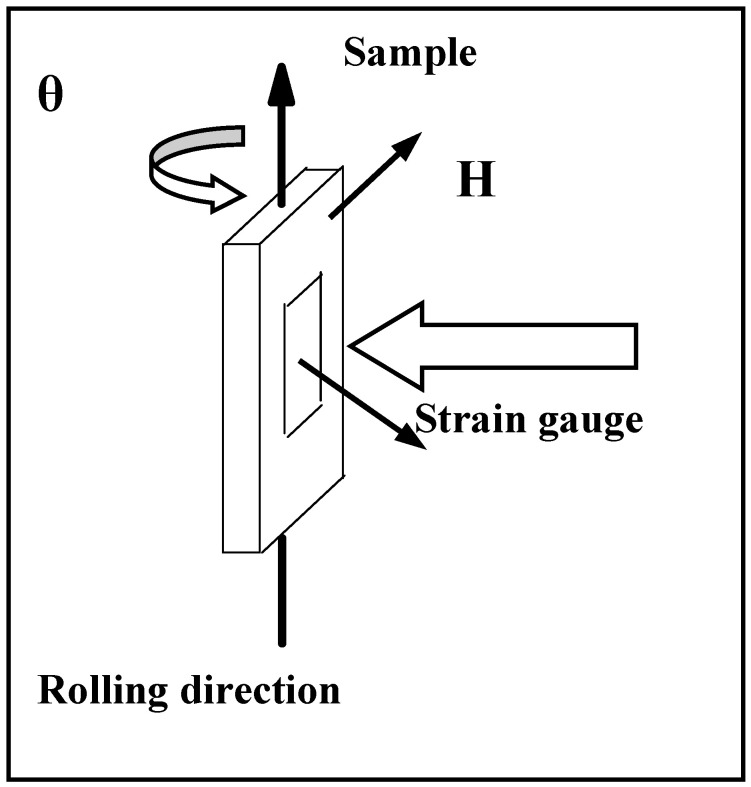
Schematics to show the setup of the strain curves measurements. The same orientation of the field H and ribbon was used for magnetic measurements.

**Figure 7:**
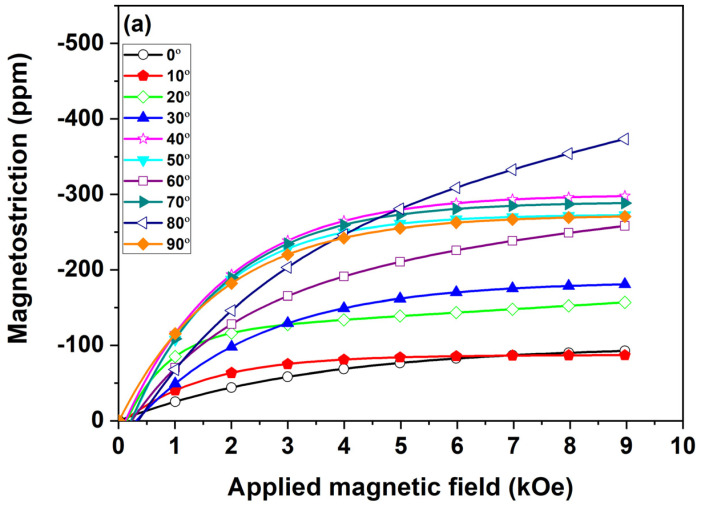

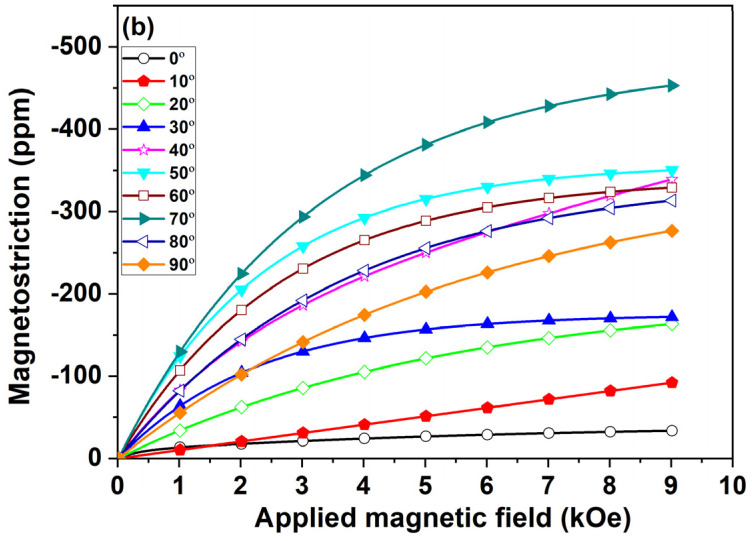
(**a**) Measured magnetoelastic strain of as-spun ribbon (S) and (**b**) annealed ribbon (A) for various angles of ribbon plane with respect to magnetic field direction.

Magnetostriction measurements for ribbons S and A were conducted electromagnetically up to 9 kOe. The ribbons were rotated to different angles with a rotating axis along the rolling direction of the melt-spinning process. The angle of rotation, θ, was defined as the angle between the ribbon surface and the field direction, as shown in Figure 6. The measured magneto-elastic deformation for ribbon S (a) and ribbon A (b) is displayed in Figure 7a,b. 

The magnetostrictive performance of ribbon S reached a maximum of 420 ppm at around 80°, whereas the minimum of about 80 ppm occurs for 0 and 10°. In this case, the magnetization rotates only in plane, and only a small effect is observed. For magnetization rotation out of plane, the magnetoelastic effect is much stronger. The magnetostriction was measured up to 9 kOe, which, for the perpendicular direction, means that the sample was only partially saturated. For the magnetostriction scales with magnetization, the maximum level of magnetostriction would be even higher. The maximum is reached only in magnetic saturation which occurs in much higher fields as indicated by the magnetization curves shown in Figure 5. 

Surprisingly, the maximum strain is not observed at 90°, but at lower angles. The probable cause of the increased magnetic strain for the 80° angle may be due to the shape anisotropy causing the ribbon to bend, especially close to the perpendicular direction even though the ribbon was sandwiched between two glass plates to prevent bending. 

A less probable, but much more desirable, cause of the increased magnetic strain may be due to the magneto-elastic effect or magnetically induced reorientation and the texture of the ribbon. The columnar grains grown along the direction of heat flow could be affected by both the wheel velocity and the turbulence of the molten alloy. Hence, the grains might be somehow tilted away from θ = 90°. These tilted columnar grains with a suitable orientation would exhibit the highest magnetically induced strain when aligned parallel to the field. However, whether the increased magneto strains are the result of the rearrangement of martensite variants due to external field remains doubtful. On the other hand, the magnetization curves for higher angle shown in Figure 5b exhibit weak but visible hysteresis, which may indicate partial variant rearrangement as in the case of Ni-Mn-Ga [3,4]. Since the difference between the lattice constants is more than 6% at room temperature (Figure 3), only fractional reorientation would be needed to increase the field-induced strain compared to magnetostriction. Further studies are needed to confirm the suggested effect. 

Surprisingly, annealed ribbon (A) consisting of bct martensite exhibits large amounts of magnetostriction too. The magnetostrictive performance of ribbon A reached a maximum of 450 ppm at around 70°, whereas the performance reached a minimum of about 30 ppm for 0° (Figure 7b). Here, the most probable cause of the increased magnetic strain for the 70° angle may be due to the shape of the anisotropy causing the ribbon to bend slightly as full bending was prevented by putting the sample between the glass plates.

## 4. Conclusions

The reversible martensitic transformation of Fe67.6-Pd32-In0.4 (at.%) melt-spun ribbon was studied in both an as-spun and annealed state. The small amount of 0.4 at.% of In added to Fe67.6-Pd32 FSMAs resulted in the increase in the transformation martensitic temperatures close to room temperature. During the transformation, tetragonality of the low temperature phase with an fct structure increased to almost 0.88 at 173 K with a consequent increase in magnetic anisotropy. Electron microscopy indicates the presence of a minor second phase with excess levels of In. However, homogenization annealing at 1273 K for 120 h resulted in an unfavoured structural change; the fully formed bct non-transforming phase was observed. Despite that, both the as-spun and the annealed ribbons exhibited large values of magnetically induced strains (over 400 ppm) within an applied field of 9 kOe at an angle of 70–80 degrees. This large field-induced strain can be used for magnetically induced modification of the layer, e.g., the ITO layer, deposited over the ribbon surface. 

## Figures and Tables

**Table 1:** The results of the composition analysis of ribbon S within and outside of the white clusters (see Figure 4).

Chemical Element	Area in the Sample					
	Within the White Clusters				Outside of the Clusters	
Pd (at.%)	37.60	34.00	34.80	39.39	30.90	32.17
In (at.%)	6.97	2.65	5.01	10.59	0.90	1.12
Fe (at.%)	55.43	63.35	59.38	50.02	68.20	66.71

## Data Availability

The data that support the findings of this study are available from the corresponding authors upon reasonable request.
